# Effect of seed morph and light level on growth and reproduction of the amphicarpic plant *Amphicarpaea edgeworthii* (Fabaceae)

**DOI:** 10.1038/srep39886

**Published:** 2017-01-10

**Authors:** Keliang Zhang, Jerry M. Baskin, Carol C. Baskin, Xuejun Yang, Zhenying Huang

**Affiliations:** 1 State Key Laboratory of Vegetation and Environmental Change, Institute of Botany, Chinese Academy of Sciences, Beijing 100093, China; 2 University of Chinese Academy of Sciences, Beijing 100039, China; 3 Department of Biology, University of Kentucky, Lexington, KY 40506, USA; 4 Department of Plant and Soil Sciences, University of Kentucky, Lexington, KY 40546, USA

## Abstract

Amphicarpic plants produce aerial and subterranean fruits on an individual plant, and these heteromorphic diaspores give rise to plants that differ in growth and ecology. *Amphicarpaea edgeworthii* is a summer annual amphicarpic species that grows over a range of light levels. We aimed to compare the response to shading intensity of plants of *A. edgeworthii* grown throughout their life cycle from aerial seeds (ASP) and from subterranean seeds (SSP). We hypothesized that vegetative and reproductive growth of plants from ASP and SSP respond differently to light. Plants were grown from ASP and SSP under 0, 46, 71 and 90% shading intensities. With plant height as a covariate, vegetative biomass of ASP and SSP did not differ. Leaf area and seed production of SSP were greater and internode length less than they were for ASP in all shading intensities. Aerial and subterranean seed yield, seed mass and number for both ASP and SSP were highest in full light. Aerial seed yield was affected more than subterranean seed yield by shading intensity. The growth and reproductive responses of ASP and SSP of *A. edgeworthii* may be adaptive to the range of low to high light environments in which this species grows.

In natural habitats, the resources necessary for plant growth such as mineral nutrients, water and light are heterogeneously distributed in space and time^1^,^2^. Plants have alternative strategies to deal with this heterogeneity, one of which is to produce seeds on the same plant that differ in morphology, mass and ecology, i.e. seed heteromorphism^3^,^4^,^5^. An extreme form of seed heteromorphism is amphicarpy, in which an individual plant produces aerial and subterranean fruits (seeds)^6^,^7^,^8^,^9^ that differ in size/mass^10^,^11^,^12^, dispersal ability^11^,^13^,^14^ and dormancy/germination^10^,^11^,^12^. Further, plants grown from aerial and subterranean seeds differ in reproductive allocation and other life history traits^15^,^16^,^17^.

Subterranean seeds of amphicarpic plants are retained in the habitat of the mother plant, where they are protected from predation, fire and desiccation^8^. Aerial seeds, on the other hand, function in colonizing new sites away from the mother plant, while subterranean seeds germinate *in situ*^6^,^12^,^14^. The subterranean seeds are usually produced earlier than the aerial ones, and this may be adaptive, because there is no guarantee that the mother plant will survive to produce aerial seeds in an unpredictable environment: the “pessimistic strategy”. In contrast, the later production of aerial seeds tends to maximize yield under favourable conditions but at the risk of not reproducing at all: the “optimistic strategy”^18^,^19^. Abiotic and biotic factors of the environment can influence the ratio of aerial and subterranean seeds produced by amphicarpic plants^9^,^11^,^16^. When resources such as light, water and nutrients are limiting for growth, the ratio of aerial : subterranean seeds is low (even zero), but under favorable conditions the ratio is high^20^,^21^,^22^. Variation in the proportion of diaspore morphs produced in different environments may enable the progeny to be more competitive under diverse environmental conditions^3^,^6^.

Light is one of the most important environmental factors affecting plant growth, and it is a key factor in the survival and growth of seedlings^23^. Spatial distribution of light can affect the spatial distribution of seedlings and determine patterns of the vegetation^24^. Further, light directly affects growth, morphology and accumulation of biomass of plants^25^. Plants growing in low light environments exhibit a series of ecological adaptations that include morphological, structural and physiological modifications, biochemical processes and gene expression^24^,^25^. Under low light, growth and development of plants are retarded, and rate of biomass accumulation and seed production decrease^25^,^26^. Analysis of species-specific responses to light conditions, such as morphology, structure and biomass allocation, can help us understand how plants are adapted to different light conditions. However, the adaptive responses of amphicarpic species to heterogeneous light conditions are not well studied. Plants of *Emex spinosa* reared from aerial seeds (ASP) and subterranean seeds (SSP) in the field grew faster under high than under low irradiance^27^, and SSP seedlings of *Polygonum thunbergii* had longer stems and greater leaf area than ASP seedlings^28^.

*Amphicarpaea edgeworthii* (Fabaceae) is an annual twining herb widely distributed in moist soils of deciduous forests, low wooded areas along streams, river banks and roadsides from full sun to dappled shade in China, India, Japan, Korea, Russia and Vietnam^29^. This species produces both aerial and subterranean seeds that differ in morphology, physiology and ecology^14^. In the field, we have observed that *A. edgeworthii* grows over a range of light levels and that the morphology of seedlings growing under these different light conditions varies considerably. With an increase in shading intensity, seedling size of both ASP and SSP decreased, and seedlings of SSP were bigger than those of ASP.

The main objective of this study was to determine the influence of shading intensity on the growth and reproduction of plants derived from aerial (ASP) and subterranean (SSP) seeds of this amphicarpic *sensu stricto* species, i.e. both flowers and seeds are produced below ground as opposed to amphicarpy *sensu lato* in which all flowers are produced above ground or at ground-level^6^. In any case, the subterranean fruits of amphicarpic *sensu stricto* plants are produced from cleistogamous flowers, whereas those of amphicarpic *senso lato* plants are produced from chasmogamous flowers^6^. In *A. edgeworthii*, subterranean seeds are produced by cleistogamous flowers and aerial seeds from chasmogamous and cleistogamous flowers^30^,^31^. Since aerial and subterranean seeds differs in morphology and ecophysiology^14^, we hypothesized that plants from the two morphs would differ in their vegetative and reproductive growth responses to light. To test this hypothesis, we compared vegetative and reproductive growth through the annual life cycle of plants produced by the two seed morphs under different shading intensities.

## Results

### Reproductive output and mass of field-collected plants from aerial and subterranean seeds

**Figure 1:**
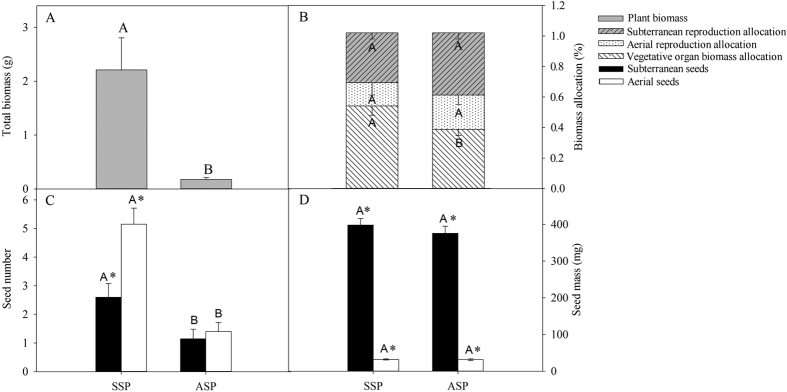
Total biomass (**A**), biomass allocation (**B**), seed number (**C**) and individual seed mass (**D**) of individual field-collected ASP and SSP *Amphicarpaea edgeworthii* plants. For each kind of measurement, different uppercase letters indicate significant difference between ASP and SSP and * indicates significant difference between aerial and subterranean seeds produced by ASP or SSP in Fig. 1C and D (5% level).

Total biomass of SSP was significantly higher than that of ASP (F = 53.628, P < 0.001; Fig. 1A). The reproductive biomass : total biomass ratio of ASP was higher than that of SSP (F = 5.527, P = 0.03), and although ASP had a higher subterranean reproductive biomass : total biomass ratio the difference was not significant (F = 0.437, P = 0.257; Fig. 1B). The number of aerial (F = 36.398, P < 0.001) and subterranean (F = 8.559, P = 0.006) seeds produced by SSP was significantly higher than that produced by ASP (Fig. 1C). However, individual mass of aerial (F = 0.067, P = 0.533) and subterranean (F = 0.290, P = 0.323) seeds of ASP and SSP was not significant (Fig. 1D).

### Effect of shading on vegetative traits

**Figure 2:**
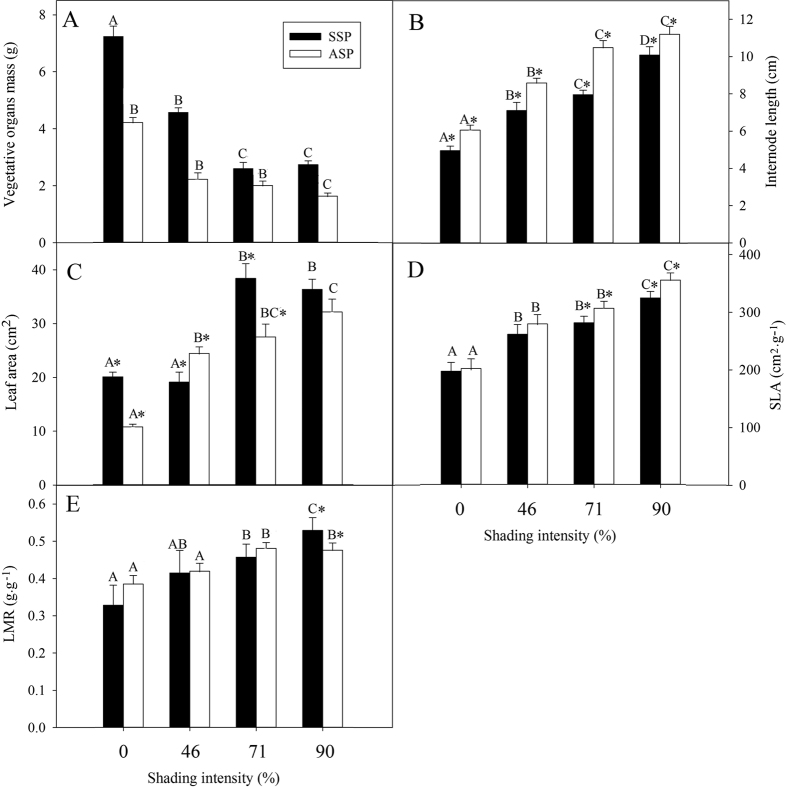
Effect of plant type (ASP or SSP) and irradiance on vegetative organ biomass (**A**), internode length (**B**), leaf area (**C**), specific leaf area (SLA, **D**) and leaf mass ratio (LMR, **E**) of *Amphicarpaea edgeworthii* plants in pot experiments. Black bars represent plants from subterranean seeds (SSP) and white bars plants from aerial seeds (ASP). For each kind of measurement, different uppercase letters indicate significant difference across all shading intensities and *indicates significant difference between ASP and SSP in the same shading intensity (5% level).

With plant height as a covariate, MANOVA tests using seed morph and shading intensity as independent variables and vegetative biomass, internode length, leaf area, SLA and LMR as dependent variables showed that both seed morph (F = 611.670, P < 0.001), shading intensity (F = 1233.948, P < 0.001) and their interaction (F = 135.140, P < 0.001) affected plant vegetative traits simultaneously. Similarly, separate tests on vegetative biomass, internode length, leaf area, SLA and LMR suggested that shading intensity and seed type had significant effects on all these characters except vegetative biomass for which the seed type effect was not significant (Table 1 and Fig. 2).

With an increase in shading intensity, vegetative biomass (Fig. 2A) decreased, and internode length (Fig. 2B), leaf area (Fig. 2C), SLA (Fig. 2D) and LMR (Fig. 2E) increased. The effect of seed type on vegetative biomass was not significant (Table 1). Leaf area (except at 46% shading) (Fig. 2C) of SSP was significantly higher than it was for ASP, but internode length (Fig. 2B) of ASP was higher than that for SSP. At 0 and 46% shading, SLA of ASP and SSP did not differ, but at 71 and 90% shading SLA of ASP was significantly greater than that of SSP (Fig. 2D). LMR of ASP and SSP did not differ significantly except at 90% shading intensity (Fig. 2E).

### Effect of shading on reproductive traits

**Figure 3:**
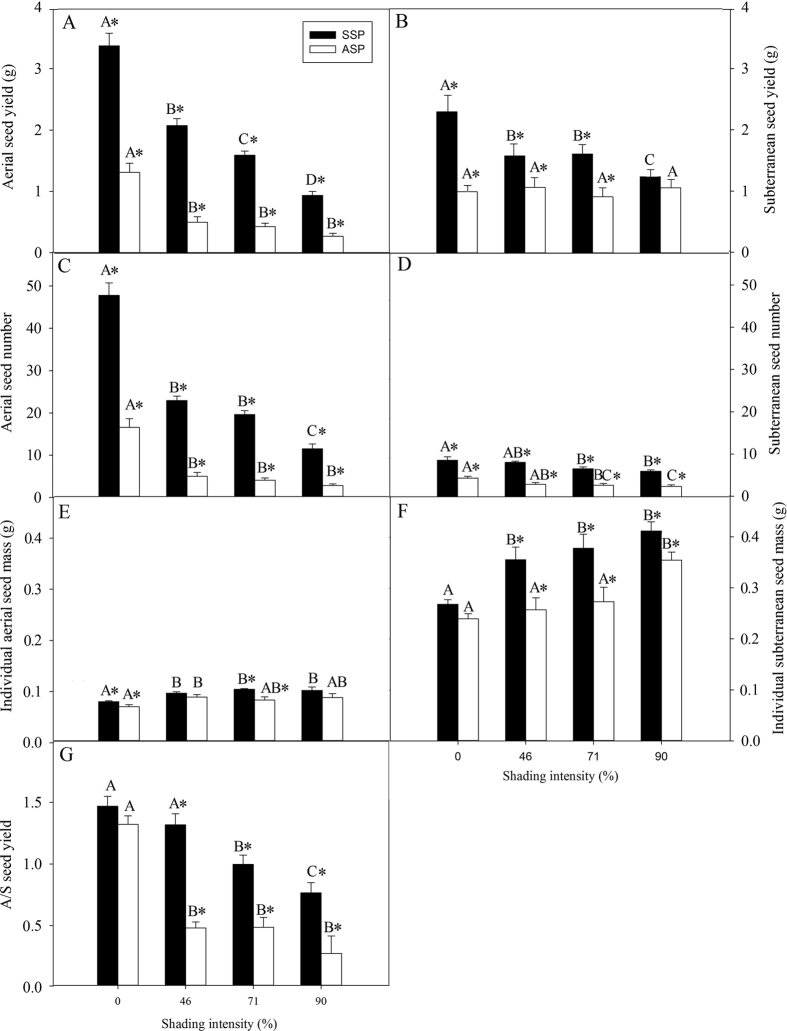
Effect of plant type (ASP or SSP) and irradiance on aerial seed yield (**A**), subterranean seed yield (**B**), aerial seed number per plant (**C**), subterranean seed number per plant (**D**), individual aerial seed mass (**E**), individual subterranean seed mass (**F**) and aerial/subterranean seed yield ratio (**G**) of plants of *Amphicarpaea edgeworthii* in pot experiments. Black bars represent plants from subterranean seeds (SSP) and white bars plants from aerial seeds (ASP). For each kind of measurement, different uppercase letters indicate significant difference across all shading intensities for the same plant type (ASP or SSP) and *indicates significant difference between ASP and SSP in the same shading intensity (5% level).

With plant height as a covariate, MANOVA tests using seed morph and shading intensity as independent variables and both aerial and subterranean seed yield, seed number, individual seed mass and A/S seed yield ratio as dependent variables showed that seed morph (F = 214.900, P < 0.001), shading intensity (F = 46.898, P < 0.001) and their interaction (F = 112.021, P < 0.001) affected all the measured reproductive traits simultaneously. Similarly, separate tests on aerial and subterranean seed yield, seed number, individual seed mass and A/S seed yield ratio suggested that shading intensity and seed type had significant effects on all these variables (Table 2 and Fig. 3).

Aerial seed yield (Fig. 3A), aerial (Fig. 3C) and subterranean (Fig. 3D) seed number and A/S seed yield ratio (Fig. 3G) of ASP and SSP decreased with an increase in shading intensity, while individual aerial (Fig. 3E) and subterranean (Fig. 3F) seed mass of ASP and SSP increased with an increase in shading intensity. Subterranean seed yield of SSP also decreased with an increase in shading, but that of ASP did not (Fig. 3B). Aerial and subterranean seed yield, seed number, individual seed mass and A/S seed yield ratio were significantly higher for SSP than for ASP.

## Discussion

Vegetative biomass of both ASP and SSP of *A. edgeworthii* decreased with an increase in shading intensity, and the trait values of untransformed variables of SSP were higher than those of ASP at all light levels. Since plants with high biomass have a competitive advantage over those with low biomass^25^, SSP should have a competitive advantage over ASP, as demonstrated for other amphicarpic species^19^,^20^,^21^,^22^,^32^,^33^. In both amphicarpic *sensu stricto* and amphicarpic *sensu lato* species, subterranean seeds generally are larger and have more mass than aerial seeds, e.g. *Amphicarpaea bracteata*^10^. *Cardamine chenopodifolia*^15^, *Catananche lutea*^32^, *Emex spinosa*^33^ and *Gymnarrhena micrantha*^20^. Thus, seedlings derived from subterranean seeds are supplied with more resources than those from aerial seeds, in which case seedlings of subterranean seeds often have a competitive advantage^15^ and exhibit higher resistance to stress^9^,^20^. For example, in *Amphicarpum purshii*, the larger subterranean seeds produce larger seedlings than the smaller aerial seeds, and large seedlings have a greater chance of survival than small ones^15^. In *A. edgeworthii*, individual aerial seed mass was 38.52 ± 0.14 mg, while individual subterranean seed mass was 456.32 ± 12.53 mg^14^. However, when plant height was used as a covariant ASP and SSP of *A. edgeworthii* did not differ significantly in biomass. Thus, the difference in untransformed biomass between ASP and SSP might be due to the different seed size. In *Polygonum thunbergii,* size of aerial and subterranean seeds was similar, and biomass allocation and total biomass of seedlings from aerial seeds and subterranean seeds did not differ with nutrient availability to the mother plant^34^.

When ASP and SSP of *A. edgeworthii* were shaded, internode length, leaf area, SLA and LMR increased. Low light severely limits photosynthesis per leaf area, and an increase in internode length, leaf area, SLA and LMR represents enhanced light interception. Thus, plants capture more light than they could have if these increases had not occurred^25^,^35^. These characteristics are well-documented shade-avoidance responses, and they are similar to those of diaspore-monomorphic herbaceous plants^36^. Internode length of ASP was significantly higher than that of SSP (Fig. 2B), while leaf area of SSP was significantly higher than that of ASP in full light and in 71% shading intensity (Fig. 2C). SLA of plants of ASP and SSP grown in either full light or 46% shading intensity did not differ significantly (Fig. 2D) and neither did LMR of plants of ASP and SSP grown in full light and in 46% and 71% shading intensity (Fig.2E). This lack of significant differences may be due to the absence of stress in these growth environments. However, with the increase in shading intensity SLA of ASP was significantly higher than that of SSP under 71% and 90% shading intensity, while LMR of SSP was significantly higher than that of ASP under 90% shading intensity. Thus, light levels apparently had different effects on vegetative growth of ASP and SSP.

Growth conditions can influence the proportion of different morphs on an individual in amphicarpic species^8^,^9^,^37^. *A. edgeworthii* produced more aerial and more subterranean seeds in full light than in shade. ASP and SSP responded differently to light in aerial and subterranean reproduction. For SSP mother plants, yield and number of aerial and subterranean seeds decreased significantly with an increase in shading intensity. For ASP mother plants, yield and number of aerial seeds decreased significantly with an increase in shading intensity. However, yield of subterranean seeds did not change, and number of subterranean seeds decreased significantly although only slightly. Seed number and yield of aerial and subterranean seeds were higher for SSP than for ASP (Fig. 3).

In amphicarpic species, production of aerial seeds generally is more plastic in response to competition and abiotic stress than that of subterranean seeds^8^. For example, in *Polygonum thunbergii* allocation to subterranean reproduction was stable at 2% under different irradiances, while aerial reproduction varied from 2% in low light intensity to 10% in high light^17^. Similar patterns were reported for *Amphicarpum purshii*^8^,^37^, *Amphicarpaea bracteata*^10^ and *Cardamine chenopodifolia*^15^. However, in *Emex spinosa* allocation to aerial reproduction increased from 7 to 46% in low to high nitrogen levels, while allocation to subterranean reproduction decreased from 38 to 3%^22^. Thus, both aerial and subterranean reproduction is quite plastic in *E. spinosa* and shows considerable adjustment to abiotic conditions^8^.

In *A. edgeworthii* and other amphicarpic species, aerial seeds have relatively high dispersal ability, while subterranean seeds remain in the vicinity of the parental plants 10,13,14,37. ASP and SSP of *A. edgeworthii* produce relative more aerial seeds in full light than subterranean seeds, and aerial seeds have a greater chance of producing new plants away from parental microsites. However, with an increase in shading intensity relative more poorly-dispersed subterranean seeds are produced, which may be important in population maintenance *in situ*. In *Amphicarpum purshii*, aerial seeds were killed by fire, but subterranean seeds were not. Therefore, seedlings present after fire were produced from subterranean seeds^16^. Thus, compared with aerial seeds the relative yield and number of subterranean seeds were higher in low than high light, and this provides an advantage to the offspring in (low light) environments that are similar to the maternal environment thereby making seedling survival more predictable. As such, production of subterranean seeds is a “timid” strategy of insuring reproduction, while production of aerial seeds is a “bold” strategy^9^.

With an increase of shading intensity SSP produced a higher A/S seed yield ratio than ASP. In 90% shading intensity, the A/S seed yield ratio was 0.26 for ASP and 0.76 for SSP. Thus, ASP is more sensitive to shading intensity than SSP, and population maintenance is more reliant on subterranean seeds and SSP than on aerial seeds and ASP. This also is the case with the amphicarpic *sensu lato* species *Emex spinosa*^22^ and the amphicarpic *sensu stricto* species *Amphicarpum purshii*^35^ and *Cardmine chenopodifolia*^15^. At densities of 15, 5 and 1 plant per pot, plants of *Amphicarpum purshii* from aerial seeds produced 0, 43 and 100% aerial spikelets, respectively, while plants from subterranean seeds produced 43, 90 and 100% aerial spikelets respectively^37^. However, in *Commelina benghalensis* grown outdoor in pots, the aerial and subterranean seed number ratio was 547.0 in ASP but only 18.3 in SSP^36^. In pots in the field, the aerial : subterranean seed number ratio for *C. benghalensis* was 81.1 in ASP and 38.7 in SSP, since ASP began tor produce seeds earlier than SSP. The high aerial and subterranean seed ratios may indicate a much greater reliance on reproduction from aerial seeds for population maintenance of *C. benghalensis* than is usual for most amphicarpic species^38^.

An increase in shading intensity resulted in an increase in individual mass of both aerial and subterranean seeds of *A. edgeworthii*, but a smaller number of seeds was produced per plant. Life history theory suggests that plants cannot increase both size and number of seeds because of resource constraints. Thus, there is a trade-off between seed number and seed mass^39^, and this trade-off affects many aspects of plant life history and even inter-specific relationships and community structure^40^. The large amount of food stored in large seeds increases the probability of survival of progeny growing in a shaded environment, while small seeds have an advantage of dispersal to diverse habitats^41^.

In summary, the reproductive strategy of *A. edgeworthii* is comparable to that of other amphicarpic annuals in that it produces a few large subterranean seeds and many small aerial seeds under good growing conditions. Also, like other amphicarpic species^10^,^24^ *A. edgeworthii* plants growing under highly adverse environmental conditions produce only subterranean seeds. For example, in 71% and 90% shading some replicates of both SSP and ASP produced subterranean seeds but no aerial seeds. Subterranean seeds remain in parental microsites, and plants resulting from them produce more aerial and subterranean seeds than plants resulting from aerial seeds in the same light environment in a mixed population of ASP and SSP under the same level of shading. This strategy may allow the species to “escape” from stressful deep shade environments via production of aerial seeds that can disperse to more favourable light microhabitats nearby. We conclude that the plasticity in the ratio of aerial and subterranean seeds produced by plants of *A. edgeworthii* grown from aerial and subterranean seeds contributes to its ability to inhabit a continuum of low to high light environments.

## Materials and Methods

### Reproductive output and mass of field-collected plants from aerial and subterranean seeds

In October 2012, when all fruits were fully mature, 20 plants each of ASP and SSP were chosen randomly in a natural habitat of *A. edgeworthii* at Fragrant Hill [Beijing, China (see Zhang *et al*.^14^ for description of site)]^14^. Germination of *A. edgeworthii* is hypogeal, and mature plants derived from ASP and SSP could be distinguished by the cotyledons that remained attached to them. Subterranean seed mass is 11.8 times greater than that of aerial seeds^14^, and thus the cotyledons of subterranean seeds are much larger than those of aerial seeds. In addition, the cotyledons of subterranean seeds usually are green, while those of aerial seeds are typically white, or absent due to decay. Roots were dug up and washed free of soil under running tap water. The number of aerial and subterranean seeds was determined for each of the 40 plants, after which whole plants were collected, put in brown paper bags and dried at 75 °C for 48 h. Whole plants were divided into vegetative parts (root, stem and leaf), aerial fruits and subterranean fruits, which were weighed using a Sartorius analytical balance (0.0001 g) after drying.

### Effect of shading on growth and reproduction

Freshly matured seeds of *A. edgeworthii* were collected on 8–14 October 2012 from a natural population of plants growing in Fragrant Hill. The desiccation tolerant aerial seeds were allowed to dry for 10 days at ambient room conditions (20–25 °C, 40–50% RH) and then stored at −18 °C in paper bags until used. The desiccation sensitive subterranean seeds were put in pots filled with natural soil (soil moisture content 14–18%) and stored at 2 °C in a refrigerator until used in experiments (see Zhang *et al*.^14^.

The shading experiment was carried out in the botanical garden of the Institute of Botany, Chinese Academy of Science, Beijing (39° 59′ 29″ N, 115° 12′ 25″ E, 65 m a. s. l). On 10 April 2013, aerial (scarified) and subterranean seeds were germinated in Petri dishes at 15/25 °C in the laboratory. On 15 April 2013, 20 seedlings (cotyledon stage) each from ASP and SSP were transplanted (one plant per pot) to each of 40 pots (21 cm in diameter and 18 cm deep) filled with soil from Fragrant Hill. Only 3-day-old seedlings of the same size were transplanted. Transplanting time was coincident with that for emergence of *A. edgeworthii* seedlings in the natural habitat at Fragrant Hill. The pots were buried in full sun in the experimental garden, so that the top was even with the soil surface.

According to measurements made using an illumination meter (HT-1300, HCJYET, GuangZhou, China) at 13 : 00 on 3 July 2013, a sunny day, plants of *A. edgeworthii* were distributed over a range of light levels from full light (0% shading) in an opening to 75.32 ± 1.87% shading under a forest canopy. Black shading-net (mesh size 2.5 mm) was used to create four shading intensities: 0% (full light), 45.72 ± 0.25% (one layer of shading-net), 71.59 ± 2.25% (two layers of shading-net) and 90.10 ± 1.85% (three layers of shading-net). Shading intensity was measured using the illumination meter described above. The light treatments were started when the seedlings had three leaves (2 May 2013, 17 days after transplanting). Before the beginning of each treatment, the soil was watered to field capacity (21.3 ± 1.5%). To prevent rainstorms from destroying the experiment, the pots were covered with transparent plastic sheeting on the rainy days of 2 July, 15 July and 12 August 2013, when the amount of precipitation was 51 mm, 48 mm and 71 mm, respectively. During the experiment, soil in the pots in all treatments was kept moist (watered daily); signs of water stress (i.e. wilting of leaves) were not observed during the experiment. Temperature and relative humidity (RH) were recorded continuously throughout the study. Mean air minimum and maximum temperatures were 11 °C and 21.8 °C, respectively, and RH was 16–73%.

Plants were harvested on 15 October 2013, when all fruits were fully mature. Roots were dug up and washed free of soil under running tap water. Each plant was divided into stems, leaves, roots and reproductive organs (including aerial and subterranean fruits). Number of leaves, plant height, internode length and number of aerial and of subterranean seeds on each plant were determined. Leaf area was scanned and analyzed using the software (WinFOLIA Pro 2004a; Regent Instruments, Québec, QC, Canada). Then, stems, leaves, roots and reproductive organs were put in brown paper bags, dried at 75 °C for 48 h and then weighted. Vegetative biomass, leaf mass ratio (LMR, g/g) and specific leaf area (SLA, cm^2^/g) were determined. Twenty randomly-chosen aerial and subterranean seeds each were weighed using the Sartorius balance.

### Data analysis

T-tests were used to compare total biomass, biomass allocation and seed number and mass of field collected ASP and SSP. Seed morph, seed size and plant size are interdependent, and it is well known that larger seeds produce larger plants, which could impact reproductive traits due to ontogentic drift^13^. To control for such possible ontogenetic effects, plant size (plant height) was used as a covariate. Thus, our null hypothesis was that there was no significant effect of plant size in the analyses caused by ontogenetic drift. A multivariate analyses (MANOVA) was used to compare the main effects of plant type, shading intensity and their interactions with vegetative biomass, internode length, leaf area, SLA, LMR and reproductive component i.e. seed yield, seed number, seed mass and A/S seed yield ratio. Wilks’ Lambda and its approximate F-values and P-values were extracted to examine the significance of seed morph and shading intensity on vegetative and reproductive traits. If MANOVA indicated significance in the data, Tukey’s HSD test was used to determine the differences between treatments (P < 0.05). Data were log_10_ transformed when necessary to improve normality and homogeneity of variances. All analyses were performed with SPSS Version 18.0 (SPSS Inc., Chicago, IL, USA).

## Additional Information

**How to cite this article**: Zhang, K. *et al*. Effect of seed morph and light level on growth and reproduction of the amphicarpic plant *Amphicarpaea edgeworthii* (Fabaceae). *Sci. Rep.* **7**, 39886; doi: 10.1038/srep39886 (2017).

**Publisher's note:** Springer Nature remains neutral with regard to jurisdictional claims in published maps and institutional affiliations.

## Figures and Tables

**Table 1:** Results of a MANOVA on effects of seed type, shading intensity and their interactions on vegetative traits of *Amphicarpaea edgeworthii*.

Source	Seed type		Shading intensity		Seed type-by-Shading intensity	
	*F*	*P*	*F*	*P*	*F*	*P*
Multivariate	1233.948	<0.001	611.670	<0.001	135.140	<0.001
Univariate						
Vegetative biomass	0.046	0.830	724.456	<0.001	132.596	<0.001
Internode length	1309.410	<0.001	609.709	<0.001	105.553	<0.001
Leaf area	83.339	<0.001	2107.963	<0.001	545.168	<0.001
SLA	1267.830	<0.001	3358.783	<0.001	185.901	<0.001
LMR	104.233	<0.001	1259.330	<0.001	18.099	<0.001

Multivariate F-statistics based on Wilks’ lambda with significance levels shown; details in Methods section.

**Table 2:** Results of a MANOVA on effects of seed type, shading intensity and their interactions on reproductive traits of *Amphicarpaea edgeworthii*.

Source	Seed type		Shading intensity		Seed type-by-Shading intensity	
	*F*	*P*	*F*	*P*	*F*	*P*
Multivariate	214.900	<0.001	46.898	<0.001	26.765	<0.001
Univariate						
Aerial seed yield (A)	575.761	<0.001	259.827	<0.001	22.369	<0.001
Subterranean seed yield (S)	654.322	<0.001	158.507	<0.001	40.822	<0.001
Aerial seed number/plant	873.821	<0.001	177.522	<0.001	57.919	<0.001
Subterranean seed number/plant	9.710	<0.001	9.759	<0.001	5.023	<0.001
Individual aerial seed mass	4.477	<0.001	9.372	<0.001	12.347	<0.001
Individual subterranean seed mass	33.723	<0.001	115.464	<0.001	5.664	<0.001
A/S seed yield ratio	436.944	<0.001	223.040	<0.001	13.520	<0.001

Multivariate F-statistics based on Wilks’ lambda with significance levels shown; details in Methods section.

## References

[b1] Jackson R. B. & Caldwell M. M. The scale of nutrient heterogeneity around individual plants and its quantification with geostatistics. Ecology 74, 612–614. (1993).

[b2] Caldwell M. M. & Pearcy R. W. Exploitation of environmental heterogeneity by plants: ecophysiological processes above and belowground. (Academic Press, San Diego, 1994).

[b3] Lloyd D. G. Variation strategies of plants in heterogeneous environments. Biol. J. Linn. Soc. 21, 357–385 (1984).

[b4] Venable D. L. The evolutionary ecology of seed heteromorphism. Am. Nat. 126, 577–595 (1985).

[b5] Yang F. . Transgenerational plasticity provides ecological diversity for a seed heteromorphic species in response to environmental heterogeneity. Perspect. Plant. Ecol. Evol. Syst. 17, 201–208 (2015).

[b6] Baskin C. C. & Baskin J. M. Seeds: ecology, biogeography, and evolution of dormancy and germination. Second edition. (Elsevier/Academic Press, San Diego, 2014).

[b7] Van der Pilj L. Principles of dispersal in higher plants. (Springer-Verlag, Berlin, 1969).

[b8] Cheplick G. P. Life history evolution in amphicarpic plants. Plant Species Biol. 9, 119–131 (1994).

[b9] Sadeh A., Guterman H., Gersani M. & Ovadia O. Plastic bet-hedging in an amphicarpic annual: an integrated strategy under variable conditions. Evol. Ecol. 23, 373–388 (2009).

[b10] Schnee B. K. & Waller D. M. Reproductive behavior of *Amphicarpaea bracteata* (Leguminosae), an amphicarpic annual. Am. J. Bot. 73, 376–386 (1986).

[b11] Ruiz de Clavijo E. & Jimenez M. J. The influence of achene type and plant density on growth and biomass allocation in the heterocarpic annual *Catananche lutea* (Asteraceae). Int. J. Plant Sci. 159, 637–647 (1998).

[b12] Kaul V., Koul A. K. & Sharma M. C. The underground flower. Curr. Sci. 78, 39–44 (2000).

[b13] Ellner S. & Shmida A. Why are adaptations for long-range seed dispersal rare in desert plants. Oecologia 51, 133–144 (1981). 10.1007/BF00344663 28310320

[b14] Zhang K. L., Baskin J. M., Baskin C. C., Yang X. J. & Huang Z. Y. Lack of divergence in seed ecology of two *Amphicarpaea* (Fabaceae) species disjunct between eastern Asia and eastern North America. Am. J. Bot. 102, 860–869 (2015). 26101412 10.3732/ajb.1500069

[b15] Cheplick G. P. Differences between plants arising from aerial and subterranean seeds in the amphicarpic annual Cardamine chenopodifolia (Cruciferae). Bull. Torrey. Bot. Club. 110, 442–448 (1983).

[b16] Cheplick G. P. & Quinn J. A. Subterranean seed production and population responses to fire in *Amphicarpum purshii* (Gramineae). J. Ecol. 76, 263–273 (1988).

[b17] Kawano S., Hara T., Hiratsuka A. & Hirota, I. Reproductive biology of an amphicarpic annual, *Polygonum thunbergii* (Polygonaceae), spatio-temporal changes in growth, structure and reproductive components of a population over an environmental gradient. Plant Species Biol. 5, 97–120 (1990).

[b18] Zeide B. Reproductive behavior of plants in time. Am. Nat. 112, 636–639 (1978).

[b19] Cheplick G. P. & Quinn J. A. *Amphicarpum purshii* and the “pessimistic strategy” in amphicarpic annuals with subterranean fruit. Oecologia 52, 327–332 (1982). 10.1007/BF00367955 28310391

[b20] Koller D. & Roth N. Studies on the ecological and physiological significance of amphicarpy in *Gymnarrhena micrantha* (Compostae). Am. J. Bot. 51, 26–35 (1964).

[b21] McNamara J. & Quinn J. A. Resource allocation and reproduction in populations of *Amphicarpum purshii* (Gramineae). Am. J. Bot. 64, 17–23 (1977).

[b22] Weiss P. W. Germination, reproduction and interference in the amphicarpic annual *Emex spinosa* (L.) Campd. Oecologia 45, 244–251 (1980). 10.1007/BF00346465 28309535

[b23] Agyeman V. K., Swaine M. D. & Thompson J. Responses of tropical forest tree seedlings to irradiance and the derivation of a light response index. J. Ecol. 87, 815–827 (1999).

[b24] Nicotra A. B., Chazdon R. L. & Iriarte S. V. B. Spatial heterogeneity of light and woody seedling regeneration in tropical wet forests. Ecology 80, 1908–1926 (1999).

[b25] Lambers H. F., Chapin S. & Thijs L. P. Plant physiological ecology. Second edition. (Springer-Verlag, New York, 2008).

[b26] Reich P. B. & Walters M. B. Leaf life-span in relation to leaf, plant and stand characteristics among diverse ecosystems. Ecol. Monogr. 62, 365–392 (1997).

[b27] Berjano R., Arista M., Talavera M., Ariza M. J. & Ortiz P. L. Plasticity and within plant sex-ratio variation in monoecious *Emex spinosa*. Turk. J. Bot. 38, 258–267 (2014).

[b28] Choo Y. H., Nam J. M., Kim J. H. & Kim J. G. Advantages of amphicarpy of *Persicaria thunbergii* in the early life history. Aquat. Bot. 121, 33–38 (2015).

[b29] Sa R. & Michael G. G. [Fabaceae] Flora of China [Wu C. Y., Raven P. H. & Hong D. Y. (eds)] 249–249 (Science Press, Beijing and Missouri Botanical Garden Press, St. Louis, 2010).

[b30] Zhang Y., Yang J. & Rao G. Y. Genetic diversity of an amphicarpic species, *Amphicarpaea edgeworthii* Benth. (Leguminosae) based on RAPD markers. Biochem. Syst. Ecol. 33, 1246–1257 (2005).

[b31] Zhang Y., Yang J. & Rao G. Y. Comparative study on the aerial and subterranean flower development in *Amphicarpaea edgeworthii* Benth. (Leguminosae: Papilionoideae), an amphicarpic species. Int. J. Plant Sci. 167, 943–949 (2006).

[b32] Ruiz de Clavijo E. The ecological significance of fruit heteromorphism in the amphicarpic species *Catananche lutea* (Asteraceae). Int. J. Plant Sci. 156, 824–833 (1995).

[b33] Evenari M., Kadouri A. & Gutterman Y. Eco-physiological investigations on the amphicarpy of *Emex spinosa* (L.) Campd. Flora. 166, 223–238 (1977).

[b34] Kim J. H., Nam J. M. & Kim J. G. Effects of nutrient availability on the amphicarpic traits of *Persicaria thunbergii*. Aquat. Bot. 131, 45–50 (2016).

[b35] Sultan S. E. & Bazzaz F. A. Phenotypic plasticity in *Polygonum persicaria*. I. Diversity and uniformity in genotypic norms of reaction to light. Evolution 47, 1009–1031 (1993). 10.1111/j.1558-5646.1993.tb02132.x 28564281

[b36] Poorter H. . Biomass allocation to leaves, stems and roots: meta-analyses of interspecific variation and environmental control. New Phytol. 193, 30–50 (2012). 22085245 10.1111/j.1469-8137.2011.03952.x

[b37] Cheplick G. P. & Quinn J. A. The shift in aerial/subterranean fruit ratio in *Amphicarpum purshii*: causes and significance. Oecologia. 57, 374–379 (1983). 10.1007/BF00377183 28309366

[b38] Walker S. R. & Evenson J. P. Biology of *Commelina benghalensis* L. in south-eastern Queensland. 1. Growth, development and seed production. Weed Res. 25, 239–244 (1985).

[b39] Smith C. C. & Fretwell S. D. The optimal balance between size and number of offspring. Am. Nat. 108, 499–506 (1974).

[b40] Hammond D. S. & Brown V. K. Seed size of woody-plants in relation to disturbance, dispersal, and soil type in wet neotropical forests. Ecology 76, 2544–2561 (1995).

[b41] Moles A. T. & Westoby M. Seed size and plant strategy across the whole life cycle. Oikos 113, 91–105 (2006).

